# Single‐cell transcriptomic atlas of different endometriosis indicating that an interaction between endometriosis‐associated mesothelial cells (EAMCs) and ectopic stromal cells may influence progesterone resistance

**DOI:** 10.1002/ctm2.70216

**Published:** 2025-02-19

**Authors:** Shengdi Hou, Jing Zhang, Zhiqiang Zhang, Hong Qu, Shuhong Li, Ying Jiang, Chongdong Liu

**Affiliations:** ^1^ Department of Obstetrics and Gynecology Capital Medical University Affiliated Beijing Chaoyang Hospital Beijing China

**Keywords:** endometriosis, epithelial–mesenchymal transition, FN1‐AKT pathway, mesothelial cells, progesterone resistance, single‐cell RNA sequence

## Abstract

**Background:**

Endometriosis is a hormone‐dependent disease, which can usually be divided into peritoneal endometriosis (PEM), deep‐infiltrating endometriosis (DIE) and ovarian endometriosis (OEM). Although the three pathologic types are essentially the same disease, they differ in pathological manifestations, molecular features, pain symptoms and hormonal responsiveness. However, there is limited literature focusing on the differences among these types. In this study, we employed single‐cell RNA sequencing (scRNA‐seq) to profile the transcriptome of each type using surgical biopsy samples obtained from six patients. We aimed to explore and elucidate the variations among these different types of endometriosis.

**Results:**

We identified five major cell types and 44 subpopulations, including the presence of mesothelial cells in all pathological types, including OEM. Furthermore, we characterised the variations in cell types across different pathological types by employing enrichment analysis to assess functions and pathways. Notably, our findings reveal distinct levels of epithelial–mesenchymal transition (EMT) processes experienced by mesothelial cells within the microenvironment of endometriotic lesions. Through ligand–receptor analysis and referencing relevant literature, we propose that mesothelial cells exert an influence on progesterone resistance in stromal cells through intercellular communication mediated by the FN1‐AKT pathway.

**Conclusions:**

Our study comprehensively characterises the heterogeneity of the different pathologic types of endometriosis and offers valuable insights into the underlying mechanisms contributing to variations in progesterone resistance across the three subtypes.

**Key points:**

- Single‐cell RNA (ScRNA) atlas across types of endometriosis is established.
- Mesothelial cells are founded in ovarian endometriosis.
- Endometriosis‐associated mesothelial cells (EAMCs) experience various level of epithelial–mesenchymal transition (EMT) process in different subtypes.
- EAMCs may exert an influence on progesterone resistance in stromal cells through intercellular communication mediated by the FN1‐AKT pathway.

## Graphical abstract

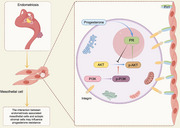

## 1 INTRODUCTION

Endometriosis is a hormone‐dependent disease in which active endometrial tissues grow and infiltrate outside the uterine cavity, affecting approximately 190 million endometriosis patients worldwide, 10% of women in reproductive age globally.^1^ Clinical manifestations of endometriosis mainly include infertility and pelvic pain, which severely affect women's quality of life.

Endometriosis mainly involves pelvic organs, and its pathologic types can be roughly categorised into: superficial peritoneal endometriosis (or peritoneal endometriosis, PEM), ovarian endometriosis (OEM) and deep‐infiltrating endometriosis (defined as endometriotic nodules with a depth of penetration exceeding 5 mm, deep‐infiltrating endometriosis, DIE). Although all three types are diagnosed by endometrial glandular and stroma, they are not identical. Nisolle and Donnez^2^ proposed that DIE lesions are adenomatous nodules, formed by remnants of Müllerian ducts that metaplasia, PEM formed by retrograde menstrual blood flow and OEM formed after epithelial invagination of the surface of the ovary, and they advocated that the three subtypes of endometriosis are three disease entities. While Konrad et al. showed that metaplasia theory had never been proved by studying the potential endometrium in Mayer‐Rokitansky‐Küster‐Hauser (MRKH) patients uterus to support Sampson's theory of endometriosis.^3^ Immunohistochemistry (IHC) demonstrated that DIE lesions had a higher level of fibrosis and underwent stronger epithelial–mesenchymal transition (EMT), fibroblast–myofibroblast transition (FMT) and smooth muscle metaplasia (SMM).^4^ Also, the use of progestogens or combined oral contraceptives for the treatment of endometriosis fails to reduce the size of the lesions of DIE compared to OEM.^5^ Above all, there is still controversy on the pathogenesis of endometriosis. However, plenty of clinical and experimental researches have suggested that types of endometriosis vary.

The treatment of endometriosis usually consists of medications and surgery.^6^ The biggest challenge in the management of endometriosis is recurrence. The influencing factors are multiple, and the related mechanisms are not fully understood, such as the unthorough surgery, EMT and progesterone resistance or intolerance. Progesterone is able to suppress inflammation levels,^7^ inhibit angiogenesis,^4^ induce apoptosis,^7^ and counteract the effects of oestrogen,^8^ thereby leading the lesion to undergo metaplasia and atrophy.^9^ Progesterone is commonly used in postoperative maintenance therapy for patients with persistent symptoms or as routine treatment for patients without fertility intention.^10^ However, progesterone therapy is ineffective in 1/4 to 1/3 of patients due to progesterone resistance or its intolerable side effects.^11^ In addition, progesterone therapy varies in its efficacy for different subtypes of endometriosis.^5^ According to the literature, response of progesterone therapy is positively related to the expression of progesterone receptors.^12^, ^13^ And PR status is significantly heterogeneous.^12^ At present, most research on progesterone resistance in endometriosis focuses on the presence of that in ectopic lesions, which shows that a lower PR expression in ectopic lesions than the eutopic, but few articles study the differences in the degree of progesterone resistance among subtypes of endometriosis. What is more, articles about endometriosis using single‐cell sequencing technique mostly focusing on the comparison between eutopic and ectopic endometrium,^14^, ^15^, ^16^ few on different subtypes of endometriosis. Therefore, further investigation of differences of endometriosis subtype, progesterone resistance and the mechanisms involved in its variability among subtypes is of great importance for disease prevention and treatment. In conclusion, though PEM, OEM and DIE are three types of endometriosis, they indeed have differences on histologic manifestations, molecular features, pain symptoms and hormonal responsiveness.

In recent years, single‐cell RNA sequencing (scRNA‐Seq) has been successfully applied to the study of the cellular components of various human organs and the heterogeneity of diseases, providing opportunities for us to understand the pathogenesis of diseases at the single‐cell level, explore the heterogeneity of diseases and solve the problems of clinical diagnosis and treatment. Tan et al. applied scRNA‐Seq technology into exploring the immune microenvironment and vascular microenvironment of endometriosis, and reported the existence of progenitor‐like epithelial cell populations in ectopic foci.^14^ The latest study by Fonseca et al. mapped the endometriotic cellular atlas in different sites and subtypes and found that the molecular characteristics of each cell population varied according to the subtypes of the tissues, suggesting the role of cellular reorganisation and transcriptional reprogramming in different subtypes of endometriosis.^17^ In our study, we will characterise the transcriptome of peritoneal, ovarian and deep‐infiltrating endometriotic lesions at the single‐cell level in patients who have not received hormone therapy any more, to explore their heterogeneity and attempt to explore the mechanisms underlying the differences in progesterone resistance among the three subtypes of endometriosis.

## 2 RESULT

### 2.1 Study population

In order to explore the relationships and differences among the three main subtypes of endometriosis at the molecular level, we performed scRNA‐seq (10X Genomics) on nine surgical biopsy samples from six patients diagnosed with endometriosis, including the different pathologic types: PEM, OEM and DIE (Supporting Information Table 1. Samples’ information). Patients without hormonal therapy before surgery will be included. Patients underwent exploratory laparoscopic surgery for ovarian cysts and had their cysts and peritoneal lesions excised, except for patient NO.6, who had no ovarian cysts detected on preoperative ultrasound or during surgery. Patient NO.6 underwent laparoscopic exploration due to intolerable dysmenorrhea, and lesions were found in Douglas cavity during surgery, which were proved to be PEM and DIE respectively. DIE1 and DIE3 samples were from the sacrouterine ligament at the time of excision of ovarian cyst. DIE2 was resected from the wall of the ovarian cyst. All samples were judged by an experienced surgeon during surgery and by a pathologist on haematoxylin and eosin (H&E)‐stained sections.

**FIGURE 1:**
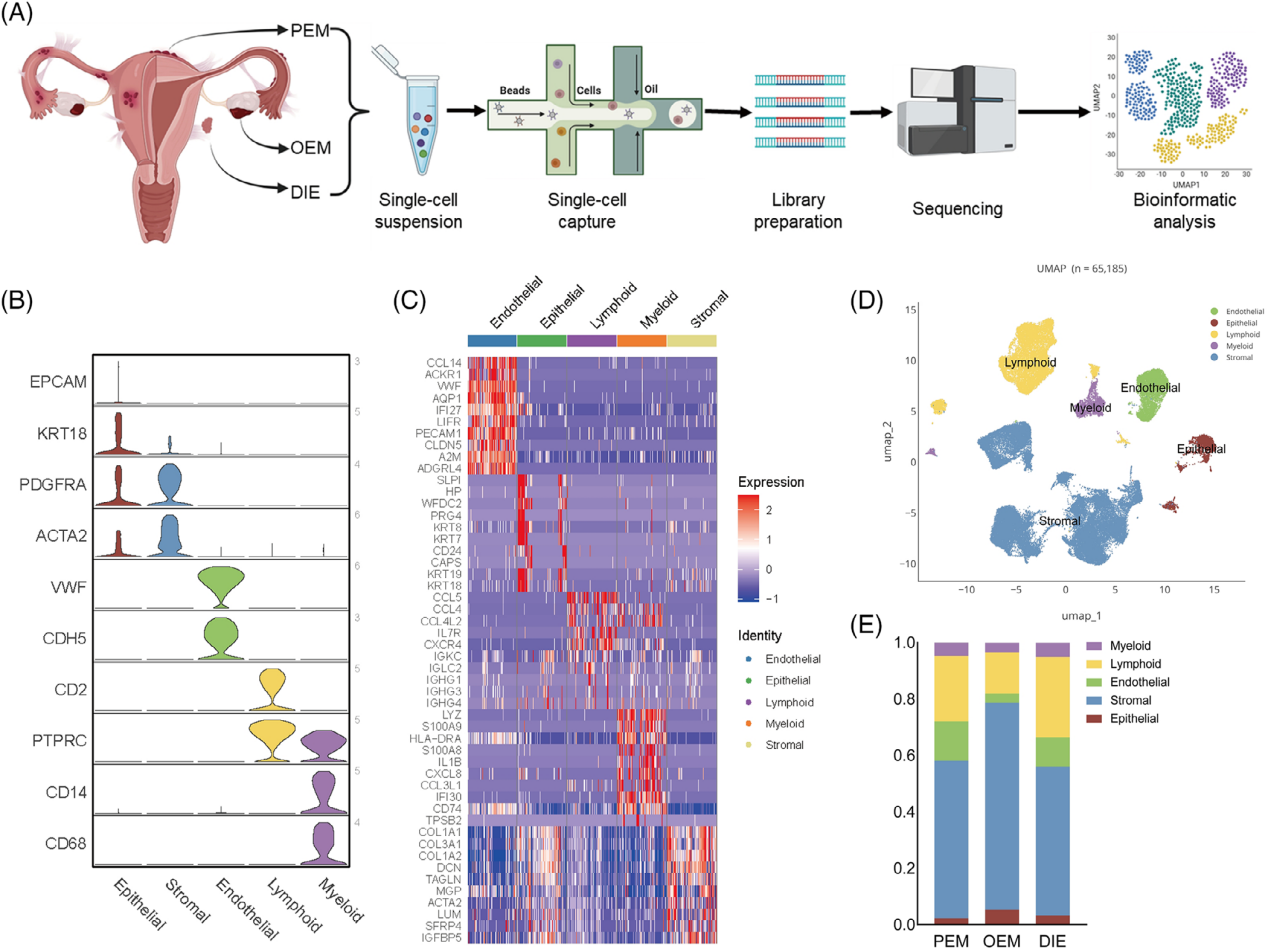
ScRNA‐transcriptomic atlas of endometriosis among different pathological types. (A) Scheme showing the collection and processing of fresh samples from three endometriosis patients for single‐cell RNA sequencing (scRNA‐seq). (B) Violin plot of marker genes expression for each cell type identified in the dataset. Cell types were classified as epithelial, stromal, endothelial, lymphoid and myeloid cells. (C) Heat map showing top‐10 marker genes of the five major clusters. (D) UMAP of 65 185 cells assigned to five major cell type according to marker genes from literatures. (E) Bar graph showing five major cell types proportions by pathological types.

After quality control as described in Section 3 (Figure S1A–C), a total of 74 643 cells from the three types of lesions were sequenced, of which 65 185 cells entered subsequent analysis (Figures 1A and S1E). DIE2 sample from the ovarian cystic wall was not included in subsequent analysis. Although it was consistent with the pathologic diagnosis of DIE. It differed significantly from the DIE group in transcriptomic similarity analysis and was similar to the OEM (Figure S1D). Perhaps some cyst wall was involved in this sample. Because the sample may be non‐classical DIE tissue, it was not included in subsequent analysis.

### 2.2 scRNA‐seq reveals multiple cellular components indifferent types of endometriosis

Unsupervised cluster analysis divided all cells into 25 clusters (Figure S1E). Based on marker genes (Figure 1B,C) in the previous literature,^14^, ^18^ cells were categorised into five cell types (Figure 1D): epithelial, stromal, endothelial, lymphoid and myeloid. Cells in each category were further to be divided into 44 subpopulations, with one cluster defined as unknown because it lacked of clearly known marker genes, demonstrating the cellular complexity of endometriosis.

When comparing the differences in the analysis of cellular component ratios among the three types at single‐cell level, mesothelial cells were excluded, for they are not an original component of the endometriotic lesion but were parts of the microenvironment. In addition, we defined an increase of more than 20% in the comparison of cell proportions as a significant cellular component change. Since both PEM and DIE lesions were located on the peritoneum, we combined them as peritoneal lesions (peritoneal ones) to compare with OEM. In this way, the heterogeneity of the cellular composition of the three pathologic types of endometriosis was evident (Figure S1G–I). Overall, more stromal cells and fewer immune cells are observed in OEM (Figure 1E). In GSVA enrichment analysis (Figure S1F), ‘interleukin‐1 receptor binding’ and terms containing ‘chemokine’, ‘fibroblast growth factor receptor’ and ‘type 2 fibroblast growth factor receptor’ GO_MF are enriched in endometriosis, which remind us that endometriosis is an inflammatory disease and closely relate to fibrosis. DIE was enriched with more ‘ferric iron binding’ and ‘type 2 fibroblast growth factor receptor’ GO_MF (Figure S1F), suggesting that DIE might associate with fibrosis and iron overload. Next, let us look at each of the cell classifications.

### 2.3 Epithelial cells

**FIGURE 2:**
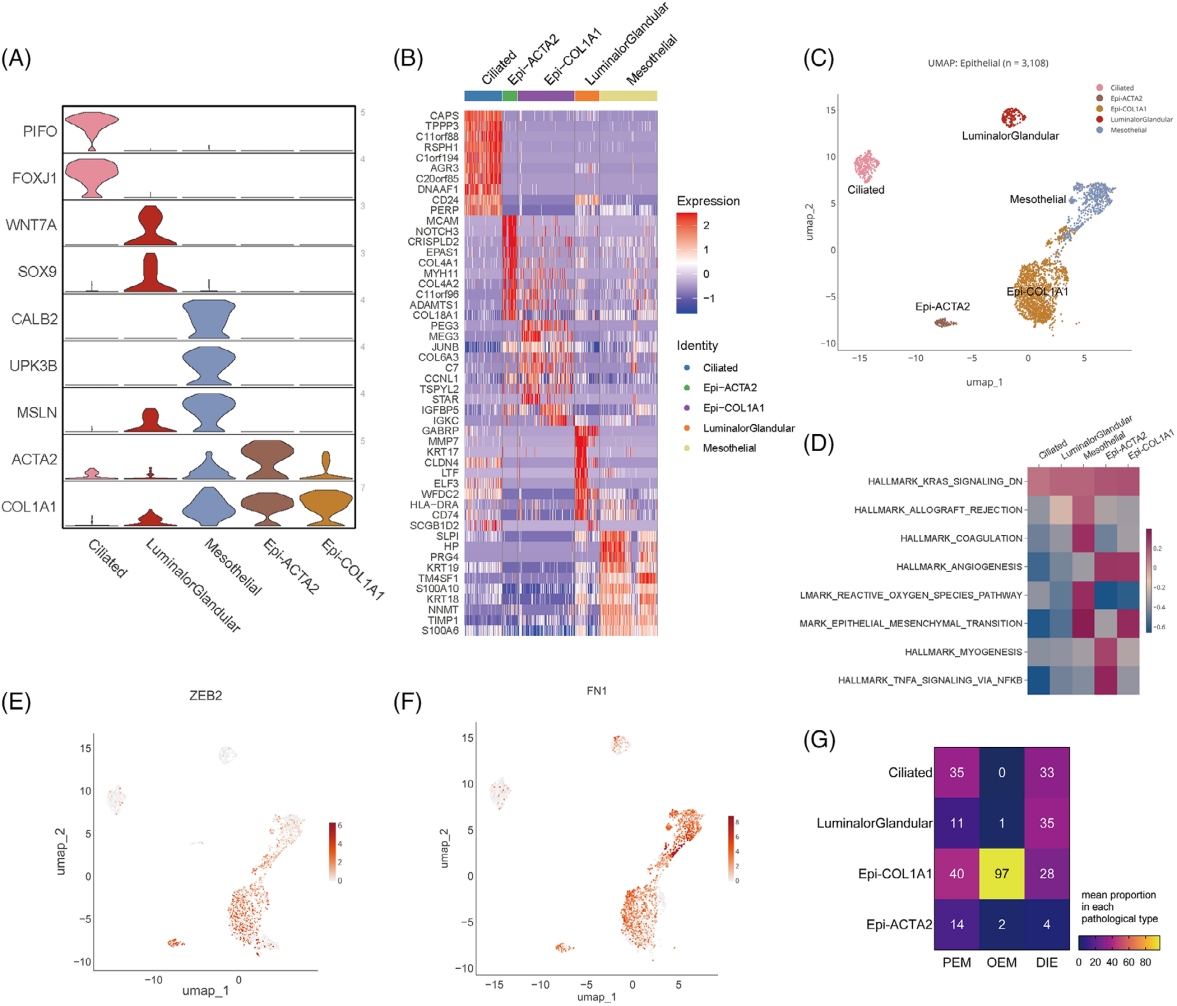
Epithelial cells. (A) Violin plot of marker genes expression for the five identified epithelial subtypes. (B) Heat map showing top‐10 marker genes of the five major clusters. (C) UMAP of epithelial subpopulations. (D) Heat map of GSVA estimating differences in hallmark activity across epithelial subtypes. GSVA scores of epithelial cells showed that these two populations associate with angiogenesis and undergo epithelial–mesenchymal transition (EMT) process. (E) ZEB2, an EMT marker, expressed in epithelial cells. (F) FN1, one of the mesenchymal marker genes, is highly expressed in Epi‐COL1A1, Epi‐ACTA2 and Mesothelial cells. (G) Heat map showing epithelial sub cell‐type proportion in each pathological type. Cell proportions are indicated as mean proportion within each square.

Histologically, endometriosis is diagnosed by the existence of ectopic stromal and epithelial cells. Firstly, we define epithelial cells by KRT18 and EPCAM (Figure 2A–C). Ciliated and luminal/glandular cells were firstly annotated in single‐cell sequencing studies of the endometrium^14^, ^17^, ^18^; however, it was not possible to differentiate between luminal and glandular epithelium in our data, probably because the number of epithelial cells was too small. In addition, two populations worth mentioning are Epi‐COL1A1 and Epi‐ACTA2, expressing fibroblast‐associated genes—COL1A1 and ACTA2 (Figure 2A). GSVA scores of epithelial cells showed that these two populations associate with angiogenesis and undergo EMT process (Figure 2D), and both of them evidently expressed ZEB2 (Figure 2E), a gene that is a marker gene in the EMT process. Above all, the two subpopulations are not classic epithelial cells, which may be experiencing EMT process and acquiring function of angiogenesis. As a result, FN1, one of the mesenchymal marker genes, is highly expressed in Epi‐COL1A1, Epi‐ACTA2 and Mesothelial cells (Figure 2F). And what confuses us is that Epi‐COL1A1 has a significantly high account in OEM lesions (Figure 2G). Thus, they deserve more and deeper researches in the future.

### 2.4 Stromal cells

**FIGURE 3:**
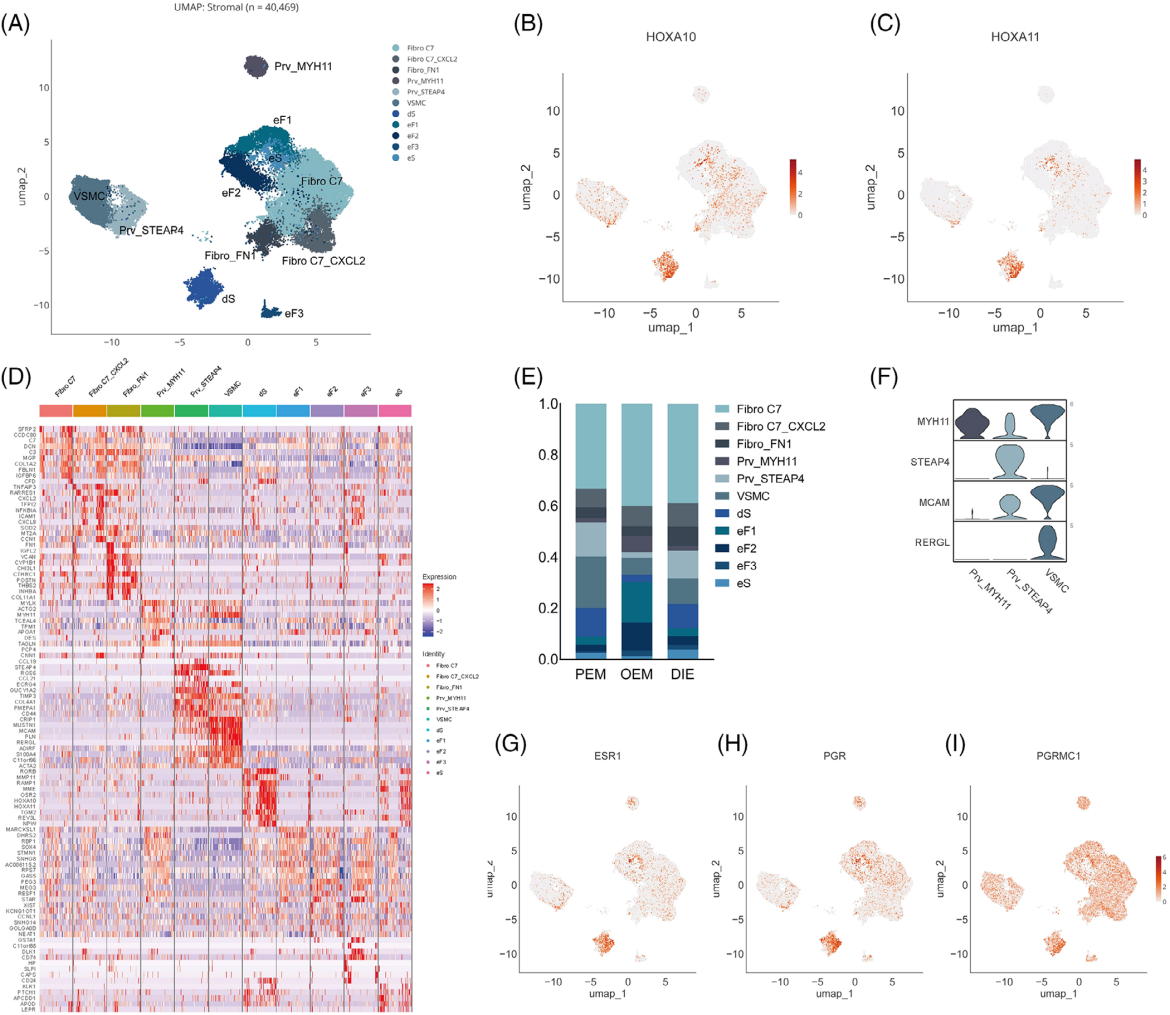
Stromal cells. (A) UMAP of stromal subpopulations. (B, C) Expression of HOXA10, HOXA11 in stromal cells. The decidualised endometrial stromal cells (dS) are characterised by the expression of HOXA10 and HOXA11. The dS subpopulation indicates that lesions may come from uterine or oviduct endometrium, supporting Sampson's theory of retrograde menstruation. (D) Heat map showing top‐10 marker genes of stromal cells. (E) Bar graph showing stromal cells proportions by pathological types. (F) Violin plot of marker genes expression for mural cells. (G–I) Expression of ESR1, PGR, PGRMC1 in stromal cells.

Overall, stromal cells are the most complex and numerous, occupying 12 clusters with a total of 40 469 cells (Figure 3A). Stromal cells can be subdivided into three major groups (Figure 3A): fibro C7, which expresses complement C7; perivascular cells, which express RGS5, MYH11, STEAP4 and RERGL (Figure 3F); and other endometrial fibroblasts, in which the eS (non‐decidualised endometrial stromal cells) and dS (decidualised endometrial stromal cells) are high in the expression of the hormone‐associated genes of ESR1, PGR and PGRMC1 (Figure 3G–I). dS are characterised by the expression of HOXA10 and HOXA11 (Figure 3B,C). The HOXA gene family is associated with the development of the Müllerian duct, and its different genes correspond to different segments of the Müllerian duct, with HOXA10 and HOXA11 corresponding to the uterine and oviduct.^19^ The dS subpopulation indicates that lesions may come from endometrium or endosalpin, supporting Sampson's theory of retrograde menstruation.

And few differences in cell component are observed in stromal cells. Fibro_FN1 was less abundant in PEM than DIE (4% vs. 8%; Figure 3E).

### 2.5 Endothelial cells

**FIGURE 4:**
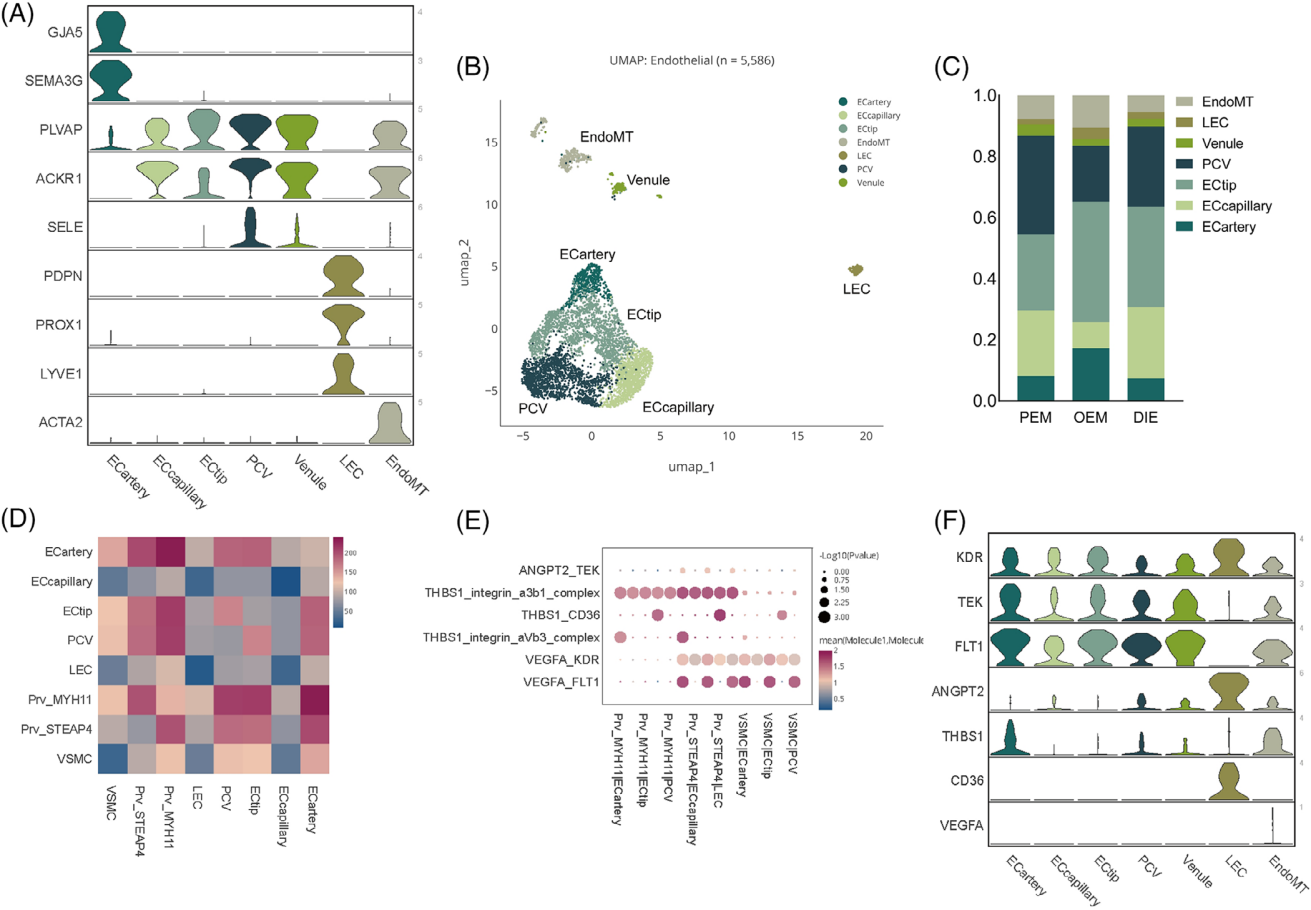
Endothelial cells. (A) Violin plot of marker genes expression for the seven identified endothelial subtypes. Endothelial cells compose a continuous vascular system with fluctuated expression of gene markers in endothelial subpopulations. (B) UMAP of endothelial subpopulations. (C) Bar graph showing endothelial proportions by pathological types. (D) Cellphone DB results revealed that pericytes interacted with endothelial cells intimately. (E) Cellphone DB analysis infer pericyte interacting with endothelial cells by ‘VEGFA_KDR’, ‘VEGFA_FLT1’, ‘THBS1_integrin_aVb3_complex’, ‘THBS1_CD36’, ‘THBS1_integrin_a3b1_complex’, ‘ANGPT2_TEK’. (F) Expression of genes participated in the interplay between pericytes and endothelial cells.

Endothelial cells, which are part of the stroma (Figure 4A,B), are independent because they characteristically express VWF and CDH5 (Figure 1B) and are a continuous vascular system with fluctuated expression of gene markers in endothelial subpopulations (Figure 4A). EndoMT co‐expresses endothelial and fibroblastic marker genes, which may suggest that it undergoes endothelial–mesenchymal transition.^20^ (Figure 4A)

Perivascular cells surround endothelial cells in capillaries and veins throughout the body. Perivascular cells (Prv_MYH11, Prv_STEAP4, VSMC) were more abundant in PEM than DIE (35% vs. 23%; Figure 4C). Perivascular cells, also called pericytes, are most in PEM among three pathological types, which interacted with endothelial cells intimately.^21^ Ligand–receptor analysis performed by Cellphone DB infer pericyte interacting with endothelial cells by ‘VEGFA_KDR’, ‘VEGFA_FLT1’, ‘THBS1_integrin_aVb3_complex’, ‘THBS1_CD36’, ‘THBS1_integrin_a3b1_complex’, ‘ANGPT2_TEK’, influencing the process of angiogenesis (Figure 4D–F). Likewise, there were significant differences in endothelial cells (Figures 1E and 4C). Endothelial cells account more in peritoneal lesions and much less in OEM. OEM contained a higher percentage of ECtip and ECartery, which is the symbol of neovascularisation.^22^ Of the relatively small number of endothelial cells in the OEM, the neoplastic endothelium nevertheless accounted for the most (Figures 1E and 4C). The reasons of this phenomenon are worth to be explored. Post capillary vessel (PCV), also called high endothelial venules, is pivotal for lymphocyte recirculation and immune surveillance,^23^ is significantly more prevalent in peritoneal lesions than in OEM (Figure 4C). PCV is an important channel for lymphocytes entering into local lymph nodes from circulation to exert their immune surveillance.^23^, ^24^ The low levels of PCV in OEM may related to ovarian immunosuppression, perhaps leading that endometriotic cysts are difficult for the immune system to eradicate and are prone to recurrence.

### 2.6 Immune cells

**FIGURE 5:**
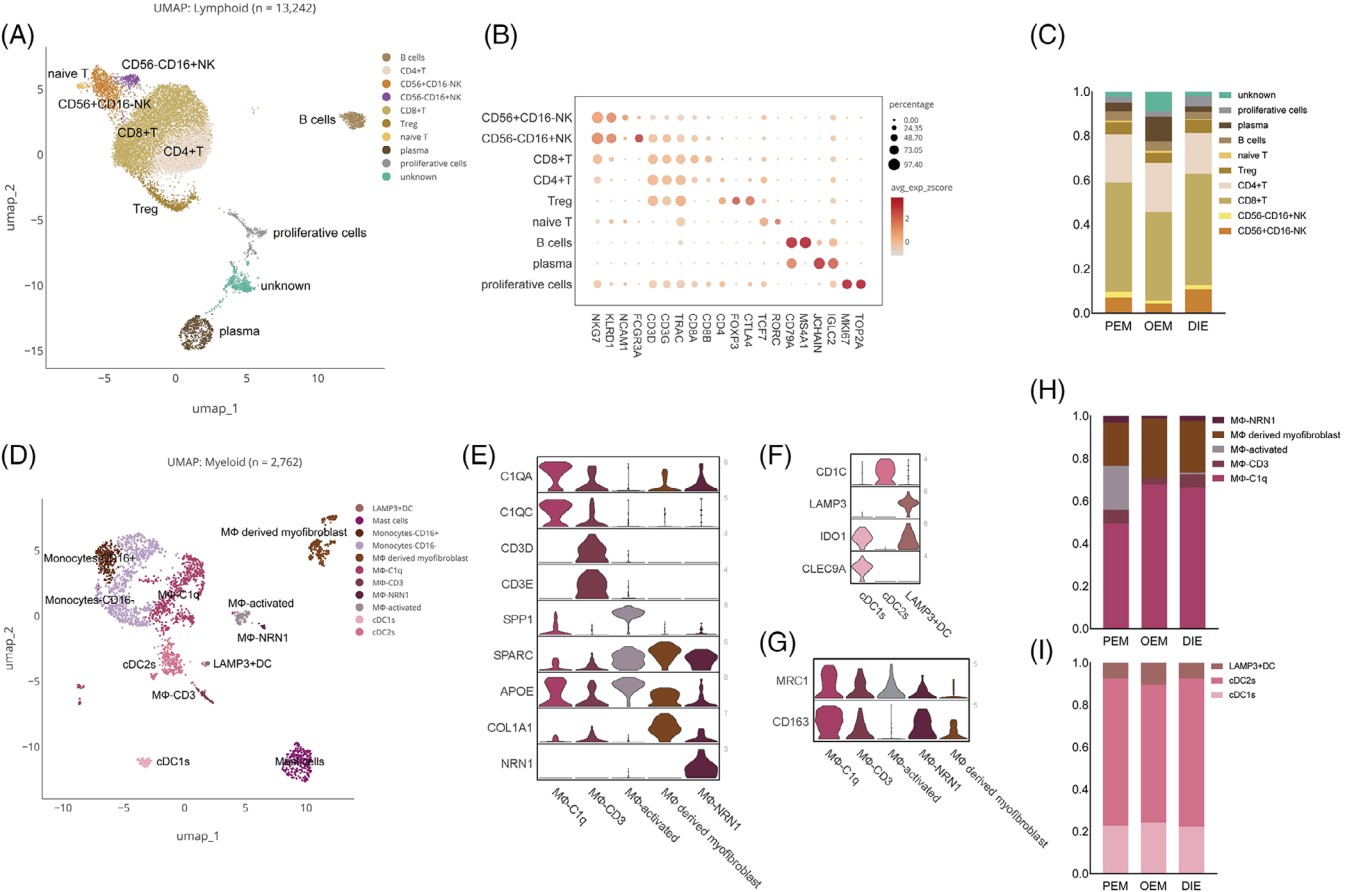
Immune cells. Immune cells can be separated into two main groups—the lymphoid lineage (A) and the myeloid lineage (D). (B) Dot plot showing maker genes for lymphoid subpopulation. (C) Bar graph showing lymphoid proportion by pathological types. (E) Violin plot showing maker genes for macrophage. (F) Marker genes of DC. (G) Violin plot of M2 marker genes expression for the macrophage subtypes. C1Q subpopulations co‐express CD163 and MRC1 (CD206). (H) Bar graph showing macrophage proportions by pathological types. (I) Bar graph showing DC proportions by pathological types.

Immune cells can be separated into two main groups—the lymphoid lineage and the myeloid lineage (Figure 5A,D). In the lymphoid lineage, we annotated T cells, B cells and proliferative cells (Figure 5A,B). Proliferative cells highly express TOP2A and MKI67, suggesting high proliferative activity (Figure 5B). The unknown population co‐expresses markers for T cells and fibroblasts (Figure 5B), which could not be annotated.

Myeloid lineage can be further continued into three major subpopulations (Figure 5D–G): DCs, referring to the annotation method and nomenclature of DCs by Heger et al.^25^ and Tan et al.^14^; monocytes; and macrophages. cDC1s and LAMP3+ DCs are likely to be immunosuppressive dendritic cells because of their expression of IDO1 (Figure 5F), and protein encoded by this gene plays a key role in inducing DC to immunosuppressive phenotype through L‐Tryptophan (Trp) metabolism via the kynurenine pathway (KP).^26^ Macrophages can be subdivided into five subpopulations (Figure 5B), revealing that macrophage was much more complex than M1/M2 dichotomy. MФ‐C1q expresses C1QA, C1QB (Figure 5B) and in either tumours or normal tissues, complement C1q is the marker of immunosuppressive macrophages.^27^ Moreover, C1Q subpopulations co‐express CD163 and MRC1 (CD206; Figure 5G), which are markers of M2 cells. In addition, MФ‐CD3, a T‐cells receptor‐positive macrophage, was again supported by our data (Figure 5B).^28^

Regarding lymphocytes, NK cells and CD8+ T cells were less in OEM, whereas plasma cells were more in OEM (Figure 5H). The difference among types may indicate an immunosuppressive microenvironment of the ovary.

### 2.7 Mesothelial cells

**FIGURE 6:**
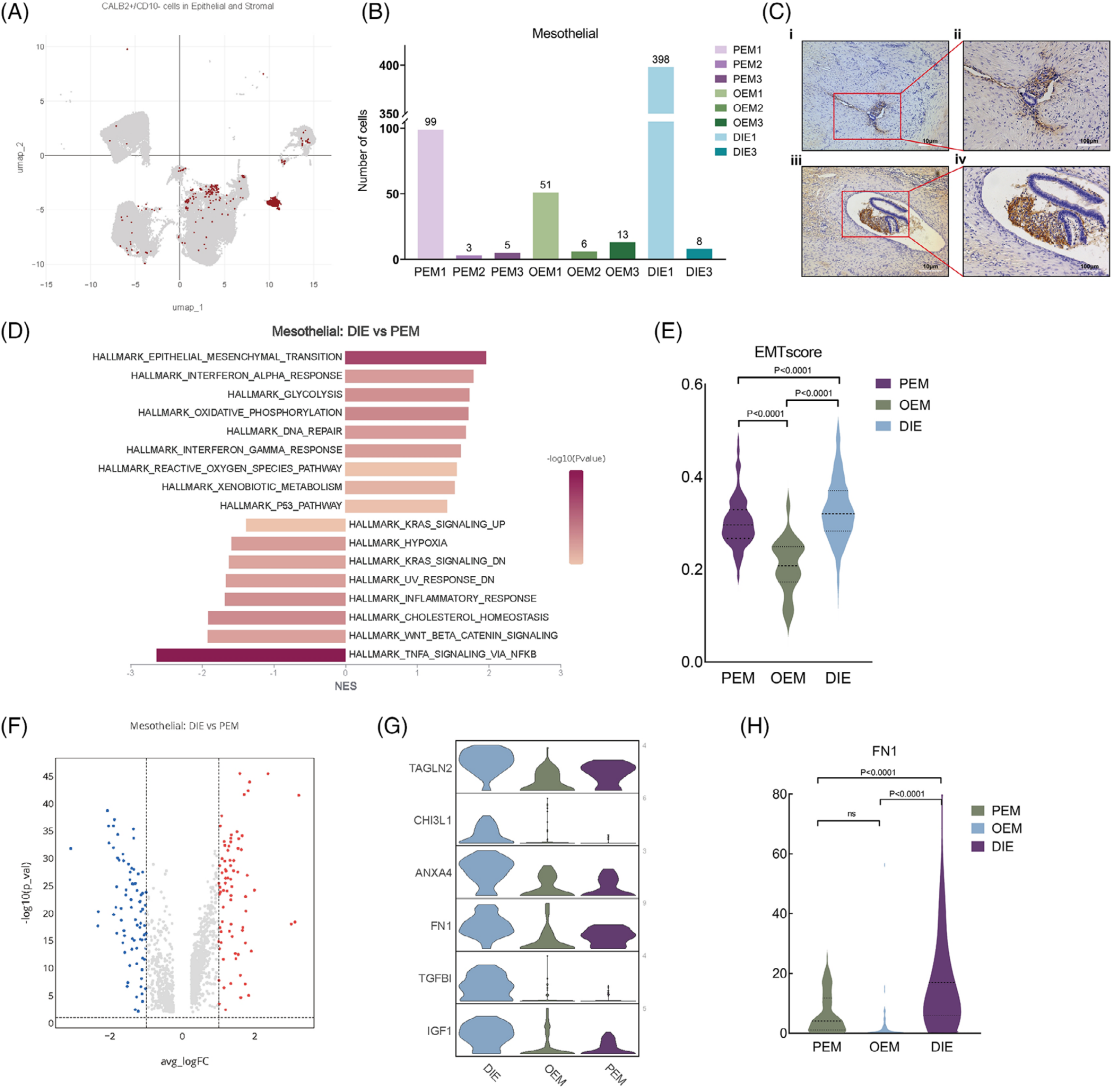
Mesothelial cells experience different level of epithelial–mesenchymal transition (EMT) process in different pathological types endometriosis. (A) We defined CALB2+/CD10− cells in epithelial and stromal cells as mesothelial cells. (B) Mesothelial cells exist in each sample of peritoneal endometriosis (PEM), deep‐infiltrating endometriosis (DIE) and even ovarian endometriosis (OEM). (C) Endometriotic cyst tissue sections were stained with the Anti‐Calretinin. Immunohistochemistry (IHC) proves that mesothelial cells exist in endometriotic cysts’ wall. (D) GSEA analysis of mesothelial cells with different pathologic types revealed that EMT‐related hallmarks were significantly upregulated in DIE compared with mesothelial cells in PEM. (E) EMT score demonstrated that mesothelial cells in DIE lesions underwent a stronger EMT process than the other two types with statistical significance. (F) Differential gene analysis on mesothelial cells between DIE and PEM, showed that 134 genes were differentially expressed in DIE mesothelial cells. (G) Several genes associated with EMT were highest in DIE samples. (H) FN1 was significantly higher in DIE than PEM or OEM at individual cell aspect.

Mesothelial cells have also been reported in previous single‐cell transcriptome sequencing articles on endometriosis.^14^, ^17^ Previous study reported that mesothelium in women with endometriosis experienced EMT,^29^, ^30^, ^31^, ^32^, ^33^ which means that mesothelial cells lose part of epithelial features and acquires characteristic typical of mesenchymal cells. According to the literatures, CALB2 is a protein expressing in mesothelial cells but not in female reproductive system cells.^34^, ^35^ Considering that mesothelial cells may experience EMT, we defined CALB2+/CD10− epithelial and stromal cells as mesothelial cells (Figure 6A), whereupon we found mesothelial cells in each pathologic type as well as in each sample (Figure 6B). For peritoneal lesions directly plant on peritoneal mesothelial cells, it is reasonable mesothelial cells exist in PEM and DIE, but we also find them in OEM, which surprises us. So, we prove that with immunohistochemistry. We apply CALB2 antibody in OEM slice, and find mesothelial cells (Figure 6C). As for the different proportion of mesothelial cells in different types of lesions, they may be related to the volume of lesions and sampling, and have less practical significance.

### 2.8 Mesothelial cells experience different level of EMT process in different pathological types of endometriosis

Since mesothelial cells exist in PEM, DIE and also OEM (Figure 6B), then we commit to exploring their similarities and discrepancies. In GSVA analysis of epithelial cells, we found that the predominant epithelial cell type in which EMT occurs is mesothelial cells, but not ectopic endometrial epithelium (Figure 2D). GSEA analysis of mesothelial cells with different pathologic types revealed that EMT‐related hallmarks were significantly upregulated in DIE mesothelial cells compared with those in PEM (Figure 6D). Also, we apply AddModuleScore function to score EMT level, demonstrating that mesothelial cells in DIE lesions underwent a stronger EMT process than the other two types with statistical significance (Figure 6E and Supporting Information Table 3. EMTscore gene. Supporting Information Table 4. AddModuleScore result–EMTscore). Then, we did differential gene analysis on mesothelial cells of DIE and PEM (Figure 6F), which showed that 159 genes were differentially expressed in DIE mesothelial cells, of which 95 were involved in EMT (Figure S3A and Supporting Information Table 2. EMT‐associated genes, https://www.genecards.org/), such as, TAGLN2, CHI3L1, FN1, IGF1, TGFBI, ANXA4, are all the highest in the DIE (Figure 6G). At the individual cell level, FN1 expression is significantly higher in mesothelial cells from DIE lesions than that from PEM and OEM (Figure 6H).

### 2.9 Endometriosis‐associated mesothelial cells interacted with ectopic endometrial stromal cells via FN1‐integrin complex

Although stromal cells are complex and heterogeneous, in the analysis of interactions between all stromal cell types and epithelial cells, we found strong interactions between each stromal cell type and mesothelial cells regardless of pathological types (Figure 3C–E), suggesting that mesothelial cells in endometriotic microenvironment may have a specific influence on the stromal components of ectopic endometrium.

**FIGURE 7:**
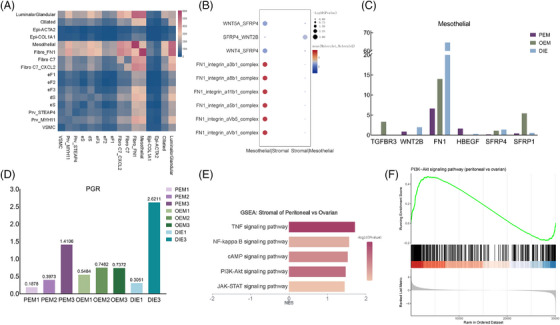
Endometriosis‐associated mesothelial cells interacted with ectopic endometrial stromal cells via FN1‐integrin complex. (A) Cellphone DB results revealed that every stomal subpopulation interacted with mesothelial cells strongly. (B) FN1_integrin_a8b1_complex, FN1_integrin_a5b1_complex, FN1_integrin_aVb5_complex and FN1_integrin_aVb1_complex FN1_integrin_a3b1_complex and FN1_integrin_a11b1_complex are determined to be the major receptor ligand pairs of mesothelial–stromal cell interactions. (C) Six genes in the ligand pairs that were both involved in epithelial–mesenchymal transition (EMT) and differentiated genes in mesothelial cells in the three types of lesions. (D) PGR has significant individual heterogeneity. (E, F) GSEA analysis indicated that PI3K‐AKT pathway was differentially activated between peritoneal and deep‐infiltrating endometriosis (DIE) lesions.

By performing ligand–receptor analysis of all epithelial and stromal cell types by CellPhone DB (Figure 7A), we found that mesothelial cells in the epithelium interacted strongly with individual stromal cells, and so we hypothesised that mesothelial cells underwent various degrees of the EMT process, which then induced various degrees of the endometriotic stromal cell progesterone resistance via AKT pathway.^36^, ^37^, ^38^, ^39^, ^40^ Between mesothelial and stromal cells, results of CellPhone DB showed a total of 268 statistically significant receptor–ligand pairs (Supporting Information Table 6. Cellphone DB results). After screening, there were a total of six genes in the above ligand pairs that were both involved in EMT and differentiated genes in mesothelial cells among the three types of lesions (Figures S3F and 7C). Of these, FN1 was the most clearly differentiated among mesothelial cells in the three types of endometriosis, both in terms of average cellular expression (Figure 7C) and in terms of expression analysis at the level of individual cells (Figure 6H). Thus, FN1_integrin_a8b1_complex, FN1_integrin_a5b1_complex, FN1_integrin_aVb5_complex, FN1_integrin_aVb1_complex, FN1_integrin_a11b1_complex and FN1_integrin_a3b1_complex are determined to be the major receptor ligand pairs of mesothelial–stromal cell interactions (Figure 7B), which may plays an important role in intercellular junctions and adhesion,^41^ inducing the initial planting of retrograde menstruation.

### 2.10 Endometriosis‐associated mesothelial cells may induce different progesterone resistance of ectopic stromal cells via FN1‐PI3K‐AKT pathway

Previous studies have shown that PGR and PR levels are significantly lower in ectopic lesions than in normal or eutopic endometrium,^5^ but there is an inconsistency in the effectiveness of oral contraceptives with progestins or oestrogen–progestogen combinations in the treatment of different types of endometriosis (Supporting Information Table 5. Progesterone efficacy in clinical practice).^5^, ^42^, ^43^, ^44^, ^45^, ^46^, ^47^, ^48^, ^49^, ^50^, ^51^, ^52^, ^53^, ^54^ In our data, PGR expression is low in stromal cells of endometriosis lesions with significant individual heterogeneity (Figure 7D). Therefore, we hypothesised that the differences in progesterone resistance were due to abnormalities in progesterone signalling. Previous studies have found that the AKT pathway,^37^, ^40^ downstream of progesterone signalling was abnormally activated in ectopic stromal cells. Meanwhile, GSEA analysis of stromal cells revealed that stromal cells from peritoneal lesions were enriched with more PI3K‐AKT pathway‐related genes than those of OEM (Figure 7E). According to a study of platinum resistant ovarian cancer,^38^ peritoneal mesothelial cells, which experience EMT process, can activated ovarian cancer cells’ AKT pathway by FN1 expression on mesothelial cells, inducing to platinum resistance. Similarly, we surmise that endometriosis‐associated mesothelial cells, which experience different EMT process and express different FN1, induced different progesterone resistance of endometrial stromal cells via the FN1‐PI3K‐AKT pathway. In order to prove this inference, we need further experiments in the future.

## 3 METHODS

### 3.1 Tissue processing

Surgical specimens were obtained with the informed consent of the patients, and the study was approved by the Ethics Committee of Capital Medical University Affiliated Beijing Chaoyang Hospital (Ethics File No. 2016‐11‐1‐2).

After surgical sampling, non‐target tissues were eliminated, blood stains were washed with saline and tissues were placed in brown sample preservation tubes containing MACS® Tissue Storage Solution (Miltenyi Biotec), transported to the laboratory at 4°C, and tissue dissociation experiments were carried out within 1 h. Tissues were removed from the sample storage tubes, washed in cooled phosphate‐buffered saline (PBS), cut into 2–3 mm pieces and dissociated for 40 min at 37°C using 3 mL of enzyme dissociation solution, which consisted of 10% fetal bovine serum (FBS) + 1 mg/mL collagenase V (Sigma: C5138)+ .1 mg DNaseI (Roche: 10104159001), and the reaction was terminated by the addition of 20 mL of Roswell Park Memorial Institute (RPMI) medium with 10% FBS after the enzyme dissociation, and then filtered through a 70 um cell sieve, and the cells were recovered by centrifugation at 4°C and 450 × *g* for 5 min. The cells were collected by centrifugation at 450 × *g* for 5 min, the supernatant was discarded and the cells were washed once using 10 mL PBS.

The undissociated tissue on the cell sieve was dissociated for 20 min at 7°C using 10 mL of enzyme dissociation solution, which consisted of .25% Trypsin EDTA + .1 mg/mL DNaseI enzyme dissociation solution. After dissociation, the reaction was terminated by the addition of 20 mL of RPMI medium with 10% FBS, filtered through a 70um cell sieve, and the cells were recovered by centrifugation at 450 × *g* for 5 min at 4°C, and the supernatant was discarded. The cells were recovered by centrifugation at 450 × *g* for 5 min at 4°C, and the supernatant was discarded.

After dissociation of collagenase and Trypsin, the cells were mixed and lysed with Red Blood Cell Lysis Solution (Miltenyi Biotec), washed once with 1× PBS and finally resuspended with PBS + .04% bovine serum albumin (BSA) + RNA inhibitor (1 U/uL), LUNA‐FL™, and LUNA‐FL™. Automated Fluorescence Cell Counter machine, AOPI dye stained the cells to detect cell activity, concentration, clumping rate and other parameters.

### 3.2 Single‐cell capture, library preparation, sequencing

Chromium Next GEM Single‐cell 3ʹ Reagent Kits v3.1 (Dual Index) kits, each sample according to 16 000 up‐sampling to complete water‐in‐oil generation, RT‐PCR amplification, cDNA amplification and library construction.

Download the kit manual at: CG000315_ChromiumNextGEMSingleCell3‐_GeneExpression_v3.1_DualIndex__RevE.pdf (ctfassets.net)

Library mass concentration was detected using Qubit 4.0 machine Qubit™ 1X dsDNA Assay Kits, high sensitivity kits, StepOnePlus™ Real‐Time PCR System for library molar concentration, LabChip Touch for library insertion fragments, illumina StepOnePlus™ Real‐Time PCR System for library molar concentration, LabChip Touch for library inserts, and illumina Novaseq6000 Sequencing Platform PE150 for read length sequencing.

### 3.3 Single‐cell data quality control and analytical processing

Illumina base call files for all libraries were demultiplexed and converted to FASTQ files using bcl2fastq v.2.20.0.422 (Illumina). Raw reads were compared to the hg38 reference genome (GRCh38 10X Genomics reference 3.0.0), matrix files were generated using cellranger 6.0.1 (10X Genomics) for raw data, and unique molecular indentifier (UMI) count matrices were processed using the R package Seurat (v.3.0.0). The resulting count matrices were further processed using the Scanpy package (v.1.7.1) to exclude genes detected in fewer than three cells and the top 2000 highly variable genes, and to exclude cells with parameters such as (1) fewer than 200 genes, (2) fewer than 1000 UMIs, (3) more than 100 000 UMIs and (4) maximum mitochondrial content of 20%. The filtered matrix was library normalised in Seurat to obtain normalised counts. Principal component analysis and neighbourhood graph generation were performed based on highly variable gene sets. Harmony (v.0.1.0) was used to minimise batch effects.

Principal component with batch effects removed was downscaled using UMAP (dimension 30, resolution .6). Differentially expressed genes in each cluster were compared to previously documented sets of cell type marker genes to annotate cell types, lineages and sub‐lineages.

Analysis were executed with functions implemented in Scanpy (1.7.1). Similarities among samples were based on hierarchical clustering calculated from Pearson correlation using the Ward linkage algorithm. In Seruat, AddModuleScore function was used to calculate the combined scores of the expression levels of a collection of different genes in each single‐cell, which are computed by averaging the expression levels of all genes in each program at the single‐cell level and subtracting the aggregated expression levels of a randomly selected set of control features. A supporting information table (Supporting Information Table 3. AddModuleScore–EMTscore gene) lists the genes used for the EMT score. EMT‐associated genes refers to https://www.genecards.org/. Gene ontology enrichment analysis and Kyoto Encyclopedia of Genes and Genomes pathway enrichment analyses were applied with the clusterProfiler in the R package for differential genes. GSEA analyses were performed by applying GSEAv.3.0 The GSEA analysis was performed using the GSEAv.3.0 software (http://software.broadinstitute.org/gsea/index.jsp).^55^, ^56^ We used the R package ‘GSVA’ for GSVA analysis.^57^, ^58^ To explore the interactions between different cell types in ectopic endometrium, we used CellPhone DB to do cell‐cell interaction analysis of all epithelial cell subpopulations and stromal cell subpopulations.^59^, ^60^

### 3.4 Immunohistochemistry

Briefly, 5‐µm thick formalin‐fixed paraffin embedded (FFPE) endometriotic tissue sections were stained with the Anti‐Calretinin (ab92341 abcam, 1:100 dilution) antibody per manufacturer's instructions at 4°C overnight after dewaxing, antigen retrieval and blocking. Antigen retrieval was performed by incubating tissue slices in citric acid buffer (pH 6.0) at 95°C for 20 min. Samples were washed and incubated with a second antibody (goat anti‐rabbit IgG H&L [HRP], Abcam: ab205718) at room temperature for 1 h. The immunohistochemistry signal was detected by 3,3′‐diaminobenzidine substrate buffer (ZLI‐9017, ZSGB‐BIO) and counter‐stained by haematoxylin (ZLI‐9610, ZSGB‐BIO). The mouse brain tissue pairs served as positive controls. All the staining results were scanned and determined by the Flexacam (Leica) scanning system in the Medical Research Center of Beijing Chaoyang Hospital.

## 4 DISCUSSION

The presented study applied single‐cell RNA sequence on a large quantity of cells across types of endometriosis, including components in endometriosis lesions, cells in immune microenvironment and the distinct mesothelial cells, mapping the single‐cell transcriptomic atlas of different types of endometriosis.

As we all know, endometriosis can be roughly divided into PEM, OEM, DIE according to lesions’ sites and infiltrating depth. Although they are diagnosed by the existence of endometriotic glandular and stroma,^1^ scholars are still arguing whether they are three disease entities,^2^ for their uncertain differences in pathogenesis, histologic manifestations, molecular features, pain symptoms and hormonal responsiveness. Unlike previous endometriosis‐associated scRNA sequencing studies,^14^ which compared eutopic endometrium and ectopic lesions, our study focuses on the slight differences among different pathological types of endometriosis.

In our study, we used CALB2 to defined mesothelial cells and use CD10 to exclude stromal cell (Figure 6A), and found them in each pathological type (Figure 6B). According to Sampson's theory,^61^ eutopic endometrium retrograde to abdominal cavity, planting on the peritoneum, then forming endometriotic lesions. In the abdominal cavity, mesothelial cells are the overlying epithelium, and are the first site contacted by the ectopic endometrium after it enters the abdominal cavity. The presence of mesothelial cells in the environment of PEM lesions is logical, but few articles have confirmed that in OEM. In our scRNA‐seq data, we found that mesothelial cells existed in each sample including all ovarian endometriotic lesions, which was further proved by IHC staining (Figure 6A–C). Therefore, we can hypothesise that OEM is mesothelial cells that wrap around the ectopic endothelial invagination to form inclusion bodies, further forming a lesion.

In the literature, it has been noted that higher levels of transforming growth factor beta (TGF‐β) in the peritoneal fluid of patients with endometriosis, leading EMT process in endometriotic lesions.^62^ Our data found that the predominant cell type undergoing EMT process was mesothelial and that its extent differed significantly among the three pathological types (Figure 6D–F). Such mesothelial cells make loss of barrier for the peritoneum, perhaps making it easier for the lesion to invade deeper leading the formation of DIE. We did differential expression analyses between pathological types, and found plenty of genes related to EMT process significantly upregulate in DIE samples. Among these genes, FN1 expression was significantly higher in mesothelial cells from DIE lesions than that from PEM and OEM, both at average expression and individual expression (Figure 6H).

As the hormonal therapy plays an important role in treatment of endometriosis, we attempt to fix something in that. Hormonal therapy is effective for symptoms but not a cure, meaning it can relieve pain without eliminating endometriotic lesions.^63^ Among them, oral contraceptives, both progestin‐only and combination, are widely used in the first‐line treatment of endometriosis, where the progestin component plays a major role.^7^ Progesterone suppresses inflammation levels, inhibits angiogenesis, induces apoptosis and counteracts the effects of oestrogens, resulting in metaplasia and atrophy of the lesion.^4^, ^7^, ^8^ Progesterone receptor expression is significantly related with response of progesterone therapy. Flores et al. have reported a 100% progesterone response rate in PR high endometriosis patients, while failed to treat PR negative patients.^12^ Progesterone resistance refers to diminished cellular responsiveness to progesterone. In the pathological state, progesterone resistance contributes to the planting of ectopic lesions outside the uterus and their ability to continue to grow and survive during the menstrual cycle. The mechanism involves high methylation of the progesterone receptor gene and abnormalities in associated transcription factors,^64^ inactivation of progesterone receptor phosphorylation,^65^ altered subcellular localisation of the progesterone receptor,^66^ impaired downstream signalling of progesterone^36^, ^39^, ^67^, ^68^, ^69^, ^70^ and abnormal metabolism of stromal cells.^71^, ^72^, ^73^ Previous studies have shown that progesterone resistance is a developmental process, which means that advanced lesions are less responsive to progesterone.

In animal experiments,^74^ compared with progesterone treatment given after modelling, the establishment, size, angiogenesis and inflammation level of ectopic lesions were more significantly suppressed when donor mice were given progesterone and then modelled. Lesions activated with progesterone signalling prior to modelling had a lesser degree of progesterone resistance, suggesting that progesterone signalling pathway is impaired in ectopic locations. Differential mRNA or protein expression of progesterone receptors in the ectopic lesions of women with or without endometriosis is controversial.^75^ Therefore, we believe that the development of progesterone resistance in endometriotic lesions is acquired, which means that progesterone resistance occurs after the endometrium component leaves the uterine cavity. Currently, most studies on progesterone resistance in endometriosis focus on the presence of that in ectopic lesions, but few articles have examined differences in the degree of progesterone resistance between endometriosis subtypes. In clinical trial, the use of progestin or oestrogen–progestin combination therapy can significantly reduce the diameter of the OEM, but not DIE (or rectovaginal interval endometriosis).^45^, ^46^, ^47^, ^48^, ^49^, ^50^, ^51^, ^52^, ^53^, ^54^, ^76^, ^77^, ^78^, ^79^ Reis et al. reviewed papers about endometriosis and progestin, reporting that DIE appear to be more resistant to size regression than PEM and OEM upon progestin therapy.^5^ Although progestin can significantly relieve the its symptoms. It cannot reduce the size of the DIE lesion, without improving the secondary complications such as hydronephrosis and intestinal obstruction. After stopping the drug, the majority of the patients' symptoms recur. That is to say, the degree of progesterone resistance still varies between subtypes. However, because of the inherent progesterone resistance of endometriosis lesions and their low progesterone receptor expression, it is difficult to compare the subtypes, and it is still controversial whether there is a difference in progesterone receptor expression in subtypes’ lesions. Therefore, we attempt to further explore the differences in progesterone resistance and mechanisms between endometriosis subtypes.

Stromal cells account a large proportion cells in each sample and is the main component of lesions, which expressing genes associated with hormone. Previous studies have found that the AKT pathway,^37^, ^40^ downstream of progesterone signalling was abnormally activated in ectopic stromal cells. In our data, AKT pathway is differently activated among different pathological types (Figure 7E). Previous literature has demonstrated that abnormal AKT activation is associated with progesterone resistance.^36^, ^39^ Increasing level of pAKT in stromal cells can abrupt decidualisation of endometriotic lesions.^80^, ^81^ On the other hand, Eaton et al. reported the inhibition of MEK1/2 or AKT pathway can increase the total and nuclear PR level in stromal cells, decreasing their viability and increasing apoptosis.^82^ It is to say that abnormal AKT activation can decrease PR protein in stromal cells, while PR expression level is positively related to progesterone response.^12^ In addition, previous study has proved that inhibition of the PI3K/Akt pathway may reverse progestin resistance in endometrial cancer by upregulating PR levels.^37^ Thus, activation of AKT pathway will lead progesterone resistance via both PR downstream and upstream pathway. But there is no study focusing on the differences among types. In our data, GSEA analysis of stromal cells revealed that stromal cells from peritoneal lesions were enriched with more PI3K‐AKT pathway‐related genes than those of OEM (Figure 7E). To some extent, it reminds us that the differences among types of responses to hormone therapy may due to different AKT pathway activation.

**FIGURE 8:**
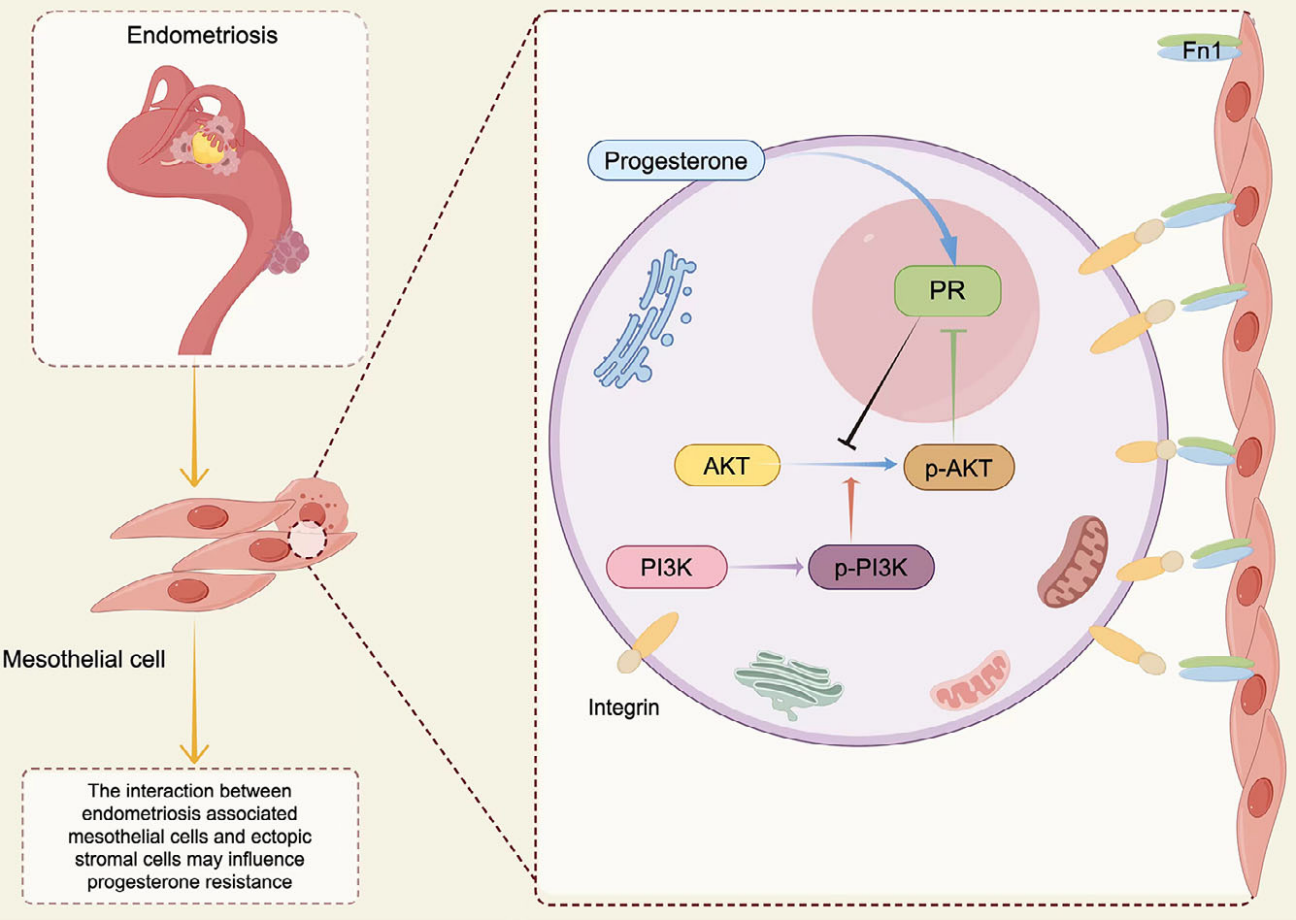
The postulated mechanism of interaction between mesothelial and stromal cells. Endometriosis‐associated mesothelial cells, experiencing different level of epithelial–mesenchymal transition (EMT) process, expressing different level of FN1, which interact directly with endometriotic stromal cells, induce a different activation of AKT pathway in stromal cells, leading to progesterone resistance.

But why is it differentially activated? Since mesothelial cells and stromal cells coexist in the microenvironment of endometriotic lesions, it is reasonable to hypothesise that they interact mutually through direct cell to cell crosstalk. We performed ligand–receptor analysis of all epithelial and stromal cell types by CellPhone DB, and found that mesothelial cells in the epithelium interacted strongly with individual stromal cells (Figure 7A). After screening, FN1 and integrin complex was determined to be the major receptor ligand pairs of mesothelial–stromal cell interactions (Figure 7B). According to a literature at ovarian cancer, which demonstrated that mesothelial cells experiencing EMT process and expressing higher level of FN1, inducing the activation of AKT pathway in cancer cells.^38^ When they cocultured the mesothelial cells which was induced to experience EMT process by TGF‐βhand the ovarian cancer cells, by blocking FN1 expression, they proved that mesothelial cells activating cancer cells AKT pathway leading to platinum resistance. Therefore, we deduce that the mesothelial cells associated with endometriosis experiencing different level of EMT process, expressing different level of FN1, which interact directly with endometriotic stromal cells, induce a different activation of AKT pathway in stromal cells, leading to progesterone resistance (Figure 8).

This present study has some limitations. Firstly, we took DIE lesions located on the ovary, but they were not included in further analyses due to the small sample size that could cause large standard errors. In the similarity analysis, it is much similar with OEM, but not DIE at transcriptome. However, we have only one DIE sample on ovarian and we did not do further explorations. In addition, our samples have few epithelial cells, which may due to the surgery or sample processing. So, we lose the analysis of epithelial cells, which may influencing the GSVA analysis result that mesothelial cells is the main part experiencing EMT. As for the interaction analysis, we only apply bioinformatics and search literature, making our deduction. In the future, our team may make more efforts in the lab exploring the interaction between mesothelial and stromal cells, to prove the importance of mesothelial cells on endometriotic progesterone resistance. We plan to use TGF‐β inducing mesothelial cells experiencing different level of EMT process. By coculturing such mesothelial cells and ectopic stromal cells, we can test the AKT and pAKT level, and when it is exposed in progesterone, decidualisation markers will be tested such as IGFBP1, PRL. By FN1 knockdown or overexpression, we can prove that it is FN1 on mesothelial cells that interact with ectopic stromal cells. Both in vitro or in vivo experiment are need in the future.

The proposed mechanism involving the FN1‐AKT pathway could serve as a basis for the development of targeted therapies to overcome progesterone resistance in endometriosis, which means that we can target the mesothelium to fix endometriotic progesterone resistance. Unlike previous studies, ours provide a novel insight to address progesterone resistance from the perspective of mesothelial cells in the microenvironment of the ectopic lesion.

## AUTHOR CONTRIBUTIONS

Shengdi Hou was responsible for the design of the experiment, data analysis, visualization, and manuscript writing. Jing Zhang was responsible for conceptualization, laboratory experimental validation, and manuscript writing. Zhiqiang Zhang, Hong Qu, Shuhong Li, and Ying Jiang were responsible for obtaining patient informed consent and providing surgical samples. Chongdong Liu was responsible for design of the program, reviewing and editing manuscript.

## CONFLICT OF INTEREST STATEMENT

The authors declare no conflicts of interest.

## ETHICS STATEMENT

Surgical specimens were obtained with the informed consent of the patients, and the study was approved by the Ethics Committee of Capital Medical University Affiliated Beijing Chaoyang Hospital (Ethics File No. 2016‐11‐1‐2). All participants were provided with information regarding the study and gave their written informed consent prior to participation. This study was conducted in compliance with the Declaration of Helsinki and all applicable ethical guidelines.

## Supporting information



Samples' information

EMT associated genes

Addmodule score‐EMT score gene

Mesothelial EMT score

Progesterone efficacy in clinical practice

Cell phone DB results

Figure S1. Quality control of scRNA‐seq. a. Column plots showing number of cells across samples and pathological types (mean ± SD). b. Violin plots indicating unique molecular identifier (UMIs) and d. gene counts per cell across samples and pathological types. d. Matrix plot representing the overall similarity of endometriosis biopsy samples from different pathological types (Pearson correlation based on gene expression from each individual). e. Uniform manifold approximation and projection (UMAP) of 65,185 cells in unsupervised cluster analysis. f. GSVA scores among different pathological types. g. h. i. UMAP of PEM, OEM, DIE samples.

Figure S2. Supplementary figure for different cell types. a. Heat map showing top‐ marker genes of lymphoid cells. b. Heat map showing top‐5 marker genes of myeloid cells. c. d. e. UMAP of lymphoid cells in PEM, OEM, DIE. f. g. h. UMAP of myeloid cells in PEM, OEM, DIE.

Figure S3. Supplementary figure for different cell types. a. 95 of the 159 DEGs involved in EMT process. (EMT associated genes refers to https://www.genecards.org/.) b. Differential gene analysis on mesothelial cells between peritoneal and ovarian lesions. c. d. e. Cellphone DB results revealed that every stomal subpopulation interacted with mesothelial cells strongly in PEM, OEM, DIE respectively. f. 6 genes in the above ligand pairs that were both involved in EMT and differentiated genes in mesothelial cells among the three types of lesions.

## References

[1] Zondervan KT , Becker CM , Missmer SA . Endometriosis. N Engl J Med. 2020;382(13):1244‐1256. doi:10.1056/NEJMra1810764 32212520

[2] Nisolle M , Donnez J . Peritoneal endometriosis, ovarian endometriosis, and adenomyotic nodules of the rectovaginal septum are three different entities. Fertil Steril. 1997;68(4):585‐596. doi:10.1016/s0015-0282(97)00191-x 9341595

[3] Konrad L , Dietze R , Kudipudi PK , Horné F , Meinhold‐Heerlein I . .Endometriosis in MRKH cases as a proof for the coelomic metaplasia hypothesis?. Reproduction. 2019;158(2):R41. doi:10.1530/rep-19-0106 30978694

[4] Liu X , Zhang Q , Guo SW . Histological and immunohistochemical characterization of the similarity and difference between ovarian endometriomas and deep infiltrating endometriosis. Reprod Sci. 2018;25(3):329‐340. doi:10.1177/1933719117718275 28718381

[5] Reis FM , Coutinho LM , Vannuccini S , Batteux F , Chapron C , Petraglia F . Progesterone receptor ligands for the treatment of endometriosis: the mechanisms behind therapeutic success and failure. Hum Reprod Update. 2020;26(4):565‐585. doi:10.1093/humupd/dmaa009 32412587 PMC7317284

[6] Taylor HS , Kotlyar AM , Flores VA . Endometriosis is a chronic systemic disease: clinical challenges and novel innovations. Lancet. 2021;397(10276):839‐852. doi:10.1016/s0140-6736(21)00389-5 33640070

[7] Reis FM , Petraglia F , Taylor RN . Endometriosis: hormone regulation and clinical consequences of chemotaxis and apoptosis. Hum Reprod Update. 2013;19(4):406‐418. doi:10.1093/humupd/dmt010 23539633 PMC3682670

[8] Jondet M , Vacher‐Lavenu MC , Chapron C . Image analysis measurements of the microvascularisation in endometrium, superficial and deep endometriotic tissues. Angiogenesis. 2006;9(4):177‐182. doi:10.1007/s10456-006-9044-y 17109198

[9] Zhang P , Wang G . Progesterone resistance in endometriosis: current evidence and putative mechanisms. Int J Mol Sci. 2023;24(8). doi:10.3390/ijms24086992 PMC10138736 37108154

[10] Zakhari A , Delpero E , McKeown S , Tomlinson G , Bougie O , Murji A . Endometriosis recurrence following post‐operative hormonal suppression: a systematic review and meta‐analysis. Hum Reprod Update. 2021;27(1):96‐107. doi:10.1093/humupd/dmaa033 33020832 PMC7781224

[11] Ferrero S , Evangelisti G , Barra F . Current and emerging treatment options for endometriosis. Expert Opin Pharmacother. 2018;19(10):1109‐1125. doi:10.1080/14656566.2018.1494154 29975553

[12] Flores VA , Vanhie A , Dang T , Taylor HS . Progesterone receptor status predicts response to progestin therapy in endometriosis. J Clin Endocrinol Metab. 2018;103(12):4561‐4568. doi:10.1210/jc.2018-01227 30357380 PMC6226602

[13] Tarumi Y , Mori T , Shimura K , et al. Progesterone receptor status of epithelial cells as a predictive marker for postoperative recurrence of endometriosis. J Clin Endocrinol Metab. 2022;107(6):1552‐1559. doi:10.1210/clinem/dgac118 35235655

[14] Tan Y , Flynn WF , Sivajothi S , et al. Single‐cell analysis of endometriosis reveals a coordinated transcriptional programme driving immunotolerance and angiogenesis across eutopic and ectopic tissues. Nat Cell Biol. 2022;24(8):1306‐1318. doi:10.1038/s41556-022-00961-5 35864314 PMC9901845

[15] Ma J , Zhang L , Zhan H , et al. Single‐cell transcriptomic analysis of endometriosis provides insights into fibroblast fates and immune cell heterogeneity. Cell Biosci. 2021;11(1):125. doi:10.1186/s13578-021-00637-x 34233737 PMC8261960

[16] Liu Z , Sun Z , Liu H , et al. Single‐cell transcriptomic analysis of eutopic endometrium and ectopic lesions of adenomyosis. Cell Biosci. 2021;11(1):51. doi:10.1186/s13578-021-00562-z 33685511 PMC7938473

[17] Fonseca MAS , Haro M , Wright KN , et al. Single‐cell transcriptomic analysis of endometriosis. Nat Genet. 2023;55(2):255‐267. doi:10.1038/s41588-022-01254-1 36624343 PMC10950360

[18] Garcia‐Alonso L , Handfield LF , Roberts K , et al. Mapping the temporal and spatial dynamics of the human endometrium in vivo and in vitro. Nat Genet. 2021;53(12):1698‐1711. doi:10.1038/s41588-021-00972-2 34857954 PMC8648563

[19] Taylor HS , Vanden Heuvel GB , Igarashi P . A conserved Hox axis in the mouse and human female reproductive system: late establishment and persistent adult expression of the Hoxa cluster genes. Biol Reprod. 1997;57(6):1338‐1345. doi:10.1095/biolreprod57.6.1338 9408238

[20] Xu Y , Kovacic JC . Endothelial to mesenchymal transition in health and disease. Annu Rev Physiol. 2023;85:245‐267. doi:10.1146/annurev-physiol-032222-080806 36266259

[21] Armulik A , Abramsson A , Betsholtz C . Endothelial/pericyte interactions. Circ Res. 2005;97(6):512‐523. doi:10.1161/01.RES.0000182903.16652.d7 16166562

[22] De Bock K , Georgiadou M , Carmeliet P . Role of endothelial cell metabolism in vessel sprouting. Cell Metab. 2013;18(5):634‐647. doi:10.1016/j.cmet.2013.08.001 23973331

[23] Blanchard L , Girard JP . High endothelial venules (HEVs) in immunity, inflammation and cancer. Angiogenesis. 2021;24(4):719‐753. doi:10.1007/s10456-021-09792-8 33956259 PMC8487881

[24] Hussain B , Kasinath V , Ashton‐Rickardt GP , et al. High endothelial venules as potential gateways for therapeutics. Trends Immunol. 2022;43(9):728‐740. doi:10.1016/j.it.2022.07.002 35931612 PMC10804419

[25] Heger L , Hofer TP , Bigley V , et al. Subsets of CD1c(+) DCs: dendritic cell versus monocyte lineage. Front Immunol. 2020;11:559166. doi:10.3389/fimmu.2020.559166 33101275 PMC7554627

[26] Platten M , Nollen EAA , Röhrig UF , Fallarino F , Opitz CA . Tryptophan metabolism as a common therapeutic target in cancer, neurodegeneration and beyond. Nat Rev Drug Discov. 2019;18(5):379‐401. doi:10.1038/s41573-019-0016-5 30760888

[27] Revel M , Sautès‐Fridman C , Fridman WH , Roumenina LT . C1q+ macrophages: passengers or drivers of cancer progression. Trends Cancer. 2022;8(7):517‐526. doi:10.1016/j.trecan.2022.02.006 35288093

[28] Chávez‐Galán L , Olleros ML , Vesin D , Garcia I . Much more than M1 and M2 macrophages, there are also CD169(+) and TCR(+) macrophages. Front Immunol. 2015;6:263. doi:10.3389/fimmu.2015.00263 26074923 PMC4443739

[29] Young VJ , Brown JK , Saunders PT , Horne AW . The role of the peritoneum in the pathogenesis of endometriosis. Hum Reprod Update. 2013;19(5):558‐569. doi:10.1093/humupd/dmt024 23720497

[30] Ono YJ , Hayashi M , Tanabe A , et al. Estradiol‐mediated hepatocyte growth factor is involved in the implantation of endometriotic cells via the mesothelial‐to‐mesenchymal transition in the peritoneum. Am J Physiol Endocrinol Metab. 2015;308(11):E950‐9. doi:10.1152/ajpendo.00573.2014 25852006

[31] Young VJ , Ahmad SF , Brown JK , Duncan WC , Horne AW . ID2 mediates the transforming growth factor‐β1‐induced Warburg‐like effect seen in the peritoneum of women with endometriosis. Mol Hum Reprod. 2016;22(9):648‐654. doi:10.1093/molehr/gaw045 27385728

[32] Young VJ , Ahmad , SF , Duncan WC , Horne AW . The role of TGF‐β in the pathophysiology of peritoneal endometriosis. Hum Reprod Update. 2017;23(5):548‐559. doi:10.1093/humupd/dmx016 28903471

[33] Yan D , Liu X , Xu H , Guo SW . Mesothelial cells participate in endometriosis fibrogenesis through platelet‐induced mesothelial‐mesenchymal transition. J Clin Endocrinol Metab. 2020;105(11):dgaa550. doi:10.1210/clinem/dgaa550 32813013

[34] Lee ES , Leong AS , Kim YS , et al. Calretinin, CD34, and alpha‐smooth muscle actin in the identification of peritoneal invasive implants of serous borderline tumors of the ovary. Mod Pathol. 2006;19(3):364‐372. doi:10.1038/modpathol.3800539 16415795

[35] Attanoos RL , Webb R , Dojcinov SD , Gibbs AR . Value of mesothelial and epithelial antibodies in distinguishing diffuse peritoneal mesothelioma in females from serous papillary carcinoma of the ovary and peritoneum. Histopathology. 2002;40(3):237‐244. doi:10.1046/j.1365-2559.2002.01352.x 11895489

[36] Kim TH , Yu Y , Luo L , Lydon JP , Jeong JW , Kim JJ . Activated AKT pathway promotes establishment of endometriosis. Endocrinology. 2014;155(5):1921‐1930. doi:10.1210/en.2013-1951 24605828 PMC3990849

[37] Gu C , Zhang Z , Yu Y , et al. Inhibiting the PI3K/Akt pathway reversed progestin resistance in endometrial cancer. Cancer Sci. 2011;102(3):557‐564. doi:10.1111/j.1349-7006.2010.01829.x 21205080 PMC11159613

[38] Yoshihara M , Kajiyama H , Yokoi A , et al. Ovarian cancer‐associated mesothelial cells induce acquired platinum‐resistance in peritoneal metastasis via the FN1/Akt signaling pathway. Int J Cancer. 2020;146(8):2268‐2280. doi:10.1002/ijc.32854 31904865 PMC7065188

[39] Yin X , Pavone ME , Lu Z , Wei J , Kim JJ . Increased activation of the PI3K/AKT pathway compromises decidualization of stromal cells from endometriosis. J Clin Endocrinol Metab. 2012;97(1):E35‐E43. doi:10.1210/jc.2011-1527 22072736 PMC3251935

[40] Cinar O , Seval Y , Uz YH , et al. Differential regulation of Akt phosphorylation in endometriosis. Reprod Biomed Online. 2009;19(6):864‐871. doi:10.1016/j.rbmo.2009.10.001 20031030

[41] Zollinger AJ , Smith ML . Fibronectin, the extracellular glue. Matrix Biol. 2017;60–61:27‐37. doi:10.1016/j.matbio.2016.07.011 27496349

[42] Muzii L , Galati G , Di Tucci C , et al. Medical treatment of ovarian endometriomas: a prospective evaluation of the effect of dienogest on ovarian reserve, cyst diameter, and associated pain. Gynecol Endocrinol. 2020;36(1):81‐83. doi:10.1080/09513590.2019.1640199 31304853

[43] Vignali M , Belloni GM , Pietropaolo G , et al. Effect of dienogest therapy on the size of the endometrioma. Gynecol Endocrinol. 2020;36(8):723‐727. doi:10.1080/09513590.2020.1725965 32065005

[44] Angioni S , Pontis A , Malune ME , et al. Is dienogest the best medical treatment for ovarian endometriomas? Results of a multicentric case control study. Gynecol Endocrinol. 2020;36(1):84‐86. doi:10.1080/09513590.2019.1640674 31311360

[45] Del Forno S , Mabrouk M , Arena A , et al. Dienogest or norethindrone acetate for the treatment of ovarian endometriomas: can we avoid surgery?. Eur J Obstet Gynecol Reprod Biol. 2019;238:120‐124. doi:10.1016/j.ejogrb.2019.04.010 31132690

[46] Angioni S , Nappi L , Pontis A , et al. A possible conservative approach in bladder endometriosis. Results of a pilot study. Gynecol Endocrinol. 2015;31(5):406‐408. doi:10.3109/09513590.2015.1006617 25776993

[47] Leonardo‐Pinto JP , Benetti‐Pinto et al. Dienogest and deep infiltrating endometriosis: the remission of symptoms is not related to endometriosis nodule remission. Eur J Obstet Gynecol Reprod Biol. 2017;211:108‐111. doi:10.1016/j.ejogrb.2017.02.015 28231497

[48] Lee JH , Song JY , Yi KW , et al. Effectiveness of dienogest for treatment of recurrent endometriosis: multicenter data. Reprod Sci. 2018;25(10):1515‐1522. doi:10.1177/1933719118779733 29848190

[49] Vercellini P , Crosignani PG , Somigliana E , Berlanda N , Barbara G , Fedele L . Medical treatment for rectovaginal endometriosis: what is the evidence?. Hum Reprod. 2009;24(10):2504‐2514. doi:10.1093/humrep/dep231 19574277

[50] Vercellini P , Buggio L , Somigliana E . Role of medical therapy in the management of deep rectovaginal endometriosis. Fertil Steril. 2017;108(6):913‐930. doi:10.1016/j.fertnstert.2017.08.038 29202965

[51] Taniguchi F , Enatsu A , Ikebuchi A , et al. Efficacy of norethisterone in patients with ovarian endometrioma. Yonago Acta Med. 2017;60(3):182‐185. 28959130 PMC5611474

[52] Park SY , Kim SH , Chae HD , Kim CH , Kang BM . Efficacy and safety of dienogest in patients with endometriosis: a single‐center observational study over 12 months. Clin Exp Reprod Med. 2016;43(4):215‐220. doi:10.5653/cerm.2016.43.4.215 28090460 PMC5234284

[53] Taniguchi F , Enatsu A , Ota I , Toda T , Arata K , Harada T . Effects of low dose oral contraceptive pill containing drospirenone/ethinylestradiol in patients with endometrioma. Eur J Obstet Gynecol Reprod Biol. 2015;191:116‐120. doi:10.1016/j.ejogrb.2015.06.006 26115056

[54] Mabrouk M , Solfrini S , Frascà C , et al. A new oral contraceptive regimen for endometriosis management: preliminary experience with 24/4‐day drospirenone/ethinylestradiol 3 mg/20 mcg. Gynecol Endocrinol. 2012;28(6):451‐454. doi:10.3109/09513590.2011.634936 22132832

[55] Wu T , Hu E , Xu S , et al. clusterProfiler 4.0: a universal enrichment tool for interpreting omics data. Innovation (Camb). 2021;2(3):100141. doi:10.1016/j.xinn.2021.100141 34557778 PMC8454663

[56] Yu G , Wang LG , Han Y , He QY . clusterProfiler: an R package for comparing biological themes among gene clusters. Omics. 2012;16(5):284‐287. doi:10.1089/omi.2011.0118 22455463 PMC3339379

[57] Hänzelmann S , Castelo R , Guinney J . GSVA: gene set variation analysis for microarray and RNA‐seq data. BMC Bioinformatics. 2013;14:7. doi:10.1186/1471-2105-14-7 23323831 PMC3618321

[58] Ritchie ME , Phipson B , Wu D , et al. limma powers differential expression analyses for RNA‐sequencing and microarray studies. Nucleic Acids Res. 2015;43(7):e47. doi:10.1093/nar/gkv007 25605792 PMC4402510

[59] Efremova M , Vento‐Tormo M , Teichmann SA , Vento‐Tormo R . CellPhoneDB: inferring cell‐cell communication from combined expression of multi‐subunit ligand‐receptor complexes. Nat Protoc. 2020;15(4):1484‐1506. doi:10.1038/s41596-020-0292-x 32103204

[60] Vento‐Tormo R , Efremova M , Botting RA , et al. Single‐cell reconstruction of the early maternal‐fetal interface in humans. Nature. 2018;563(7731):347‐353. doi:10.1038/s41586-018-0698-6 30429548 PMC7612850

[61] Sampson JA . Metastatic or embolic endometriosis, due to the menstrual dissemination of endometrial tissue into the venous circulation. Am J Pathol. 1927;3(2):93‐110.43. 19969738 PMC1931779

[62] Sandoval P , Jiménez‐Heffernan JA , Guerra‐Azcona G , et al. Mesothelial‐to‐mesenchymal transition in the pathogenesis of post‐surgical peritoneal adhesions. J Pathol. 2016;239(1):48‐59. doi:10.1002/path.4695 27071481

[63] Chapron C , Marcellin L , Borghese B , Santulli P . Rethinking mechanisms, diagnosis and management of endometriosis. Nat Rev Endocrinol. 2019;15(11):666‐682. doi:10.1038/s41574-019-0245-z 31488888

[64] Wu Y , Strawn E , Basir Z , Halverson G , Guo SW . Promoter hypermethylation of progesterone receptor isoform B (PR‐B) in endometriosis. Epigenetics. 2006;1(2):106‐111. doi:10.4161/epi.1.2.2766 17965625

[65] Wu Y , Starzinski‐Powitz A , Guo SW . Prolonged stimulation with tumor necrosis factor‐alpha induced partial methylation at PR‐B promoter in immortalized epithelial‐like endometriotic cells. Fertil Steril. 2008;90(1):234‐237. doi:10.1016/j.fertnstert.2007.06.008 17727850

[66] Yilmaz BD , Bulun SE . Endometriosis and nuclear receptors. Hum Reprod Update. 2019;25(4):473‐485. doi:10.1093/humupd/dmz005 30809650 PMC6601390

[67] Marquardt RM , Kim TH , Shin JH , Jeong JW . Progesterone and estrogen signaling in the endometrium: what goes wrong in endometriosis?. Int J Mol Sci. 2019;20(15):3822. doi:10.3390/ijms20153822 31387263 PMC6695957

[68] Pabona JM , Simmen FA , Nikiforov MA , et al. Krüppel‐like factor 9 and progesterone receptor coregulation of decidualizing endometrial stromal cells: implications for the pathogenesis of endometriosis. J Clin Endocrinol Metab. 2012;97(3):E376‐E392. doi:10.1210/jc.2011-2562 22259059 PMC3319212

[69] Hirota Y , Tranguch S , Daikoku T , et al. Deficiency of immunophilin FKBP52 promotes endometriosis. Am J Pathol. 2008;173(6):1747‐1757. doi:10.2353/ajpath.2008.080527 18988805 PMC2626386

[70] Tranguch S , Wang H , Daikoku T , Xie H , Smith DF , Dey SK . FKBP52 deficiency‐conferred uterine progesterone resistance is genetic background and pregnancy stage specific. J Clin Invest. 2007;117(7):1824‐1834. doi:10.1172/jci31622 17571166 PMC1888571

[71] Cheng YH , Imir A , Fenkci V , Yilmaz MB , Bulun SE . Stromal cells of endometriosis fail to produce paracrine factors that induce epithelial 17beta‐hydroxysteroid dehydrogenase type 2 gene and its transcriptional regulator Sp1: a mechanism for defective estradiol metabolism. Am J Obstet Gynecol. 2007;196(4):391.e1‐7. doi:10.1016/j.ajog.2006.12.014. discussion 391.e7‐8. 17403431

[72] Pavone ME , Dyson M , Reirstad S , et al. Endometriosis expresses a molecular pattern consistent with decreased retinoid uptake, metabolism and action. Hum Reprod. 2011;26(8):2157‐2164. doi:10.1093/humrep/der172 21659316 PMC3137392

[73] Pavone ME , Reierstad S , Sun H , Milad M , Bulun SE , Cheng YH . Altered retinoid uptake and action contributes to cell survival in endometriosis. J Clin Endocrinol Metab. 2010;95(11):E300‐9. doi:10.1210/jc.2010-0459 20702525 PMC2968735

[74] Li Y , Adur MK , Kannan A , et al. Progesterone alleviates endometriosis via inhibition of uterine cell proliferation, inflammation and angiogenesis in an immunocompetent mouse model. PLoS One. 2016;11(10):e0165347. doi:10.1371/journal.pone.0165347 27776183 PMC5077092

[75] McKinnon B , Mueller M , Montgomery G . Progesterone resistance in endometriosis: an acquired property?. Trends Endocrinol Metab. 2018;29(8):535‐548. doi:10.1016/j.tem.2018.05.006 29934050

[76] Minelli L , Fanfani F , Fagotti A , et al. Laparoscopic colorectal resection for bowel endometriosis: feasibility, complications, and clinical outcome. Arch Surg. 2009;144(3):234‐239. doi:10.1001/archsurg.2008.555. discussion 239. 19289662

[77] Kruse C , Seyer‐Hansen M , Forman A . Diagnosis and treatment of rectovaginal endometriosis: an overview. Acta Obstet Gynecol Scand. 2012;91(6):648‐657. doi:10.1111/j.1600-0412.2012.01367.x 22268648

[78] Vercellini P , Buggio L , Berlanda N , Barbara G , Somigliana E , Bosari S . Estrogen‐progestins and progestins for the management of endometriosis. Fertil Steril. 2016;106(7):1552‐1571. doi:10.1016/j.fertnstert.2016.10.022 27817837

[79] Lee SR , Yi KW , Song JY , et al. Efficacy and safety of long‐term use of dienogest in women with ovarian endometrioma. Reprod Sci. 2018;25(3):341‐346. doi:10.1177/1933719117725820 29161960

[80] Yoshino O , Osuga Y , Hirota Y , et al. Akt as a possible intracellular mediator for decidualization in human endometrial stromal cells. Mol Hum Reprod. 2003;9(5):265‐269. doi:10.1093/molehr/gag035 12728019

[81] Lathi RB , Hess AP , Tulac S , Nayak NR , Conti M , Giudice LC . Dose‐dependent insulin regulation of insulin‐like growth factor binding protein‐1 in human endometrial stromal cells is mediated by distinct signaling pathways. J Clin Endocrinol Metab. 2005;90(3):1599‐1606. doi:10.1210/jc.2004-1676 15613433

[82] Eaton JL , Unno K , Caraveo M , Lu Z , Kim JJ . Increased AKT or MEK1/2 activity influences progesterone receptor levels and localization in endometriosis. J Clin Endocrinol Metab. 2013;98(12):E1871‐E1879. doi:10.1210/jc.2013-1661 24064688 PMC3849681

