# Supplementary figures and images for: Single‐cell transcriptomic atlas of different endometriosis indicating that an interaction between endometriosis‐associated mesothelial cells (EAMCs) and ectopic stromal cells may influence progesterone resistance
Source: Clin Transl Med. 2025 Feb 19;15(2):e70216. doi: 10.1002/ctm2.70216 (PMC11836620; doi:10.1002/ctm2.70216)

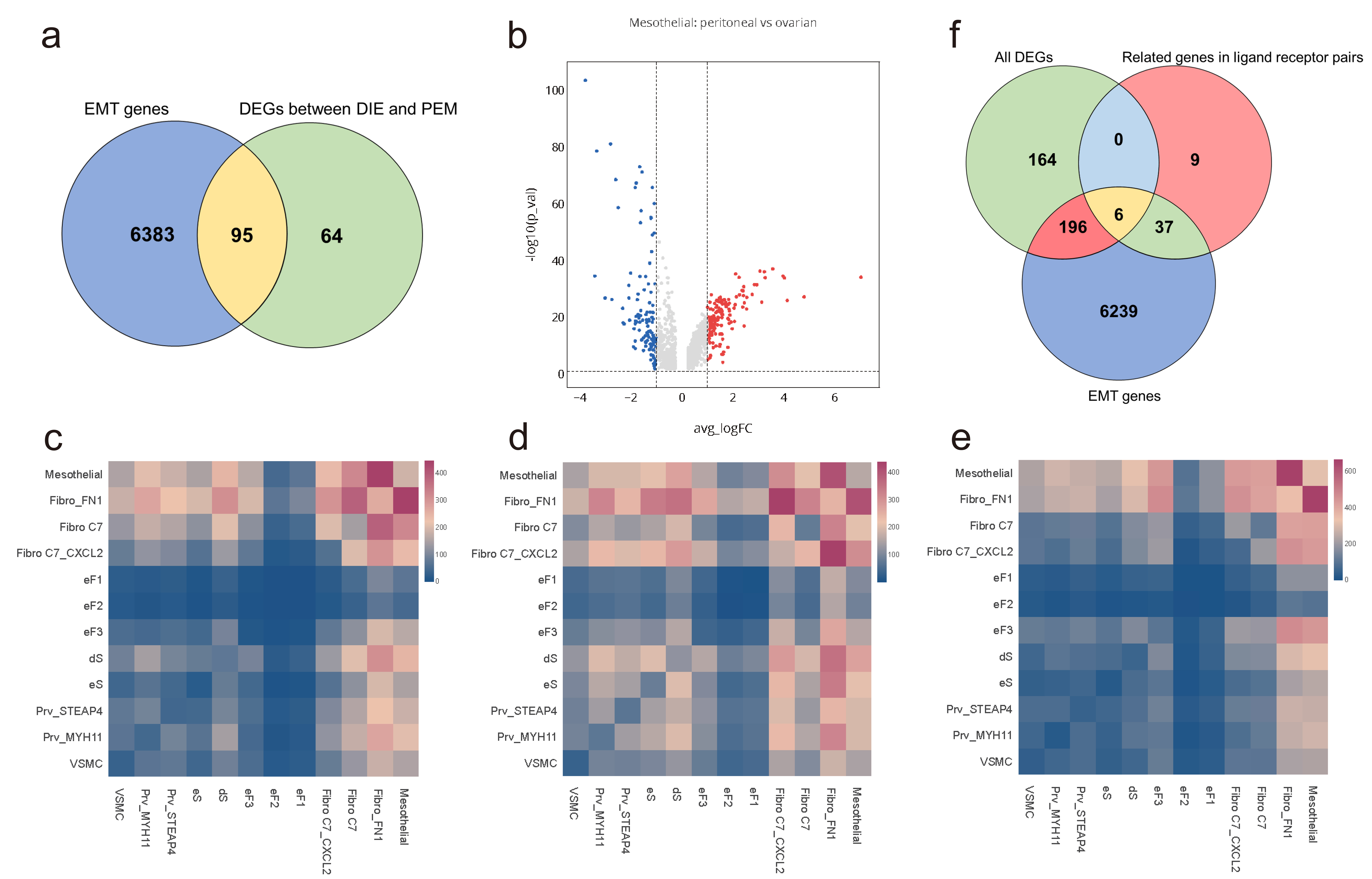

Supplement: Supplementary file 8 — Figure S2. Supplementary figure for different cell types. a. Heat map showing top‐ marker genes of lymphoid cells. b. Heat map showing top‐5 marker genes of myeloid cells. c. d. e. UMAP of lymphoid cells in PEM, OEM, DIE. f. g. h. UMAP of myeloid cells in PEM, OEM, DIE. [file CTM2-15-e70216-s002.jpg]
